# Psychiatric disorders in children with 16p11.2 deletion and duplication

**DOI:** 10.1038/s41398-018-0339-8

**Published:** 2019-01-16

**Authors:** Maria Niarchou, Samuel J. R. A. Chawner, Joanne L. Doherty, Anne M. Maillard, Sébastien Jacquemont, Wendy K. Chung, LeeAnne Green-Snyder, Raphael A. Bernier, Robin P. Goin-Kochel, Ellen Hanson, David E. J. Linden, Stefanie C. Linden, F. Lucy Raymond, David Skuse, Jeremy Hall, Michael J. Owen, Marianne B. M. van den Bree

**Affiliations:** 1 Division of Psychological Medicine and Clinical Neurosciences, Medical Research Council Centre for Neuropsychiatric Genetics and Genomics, Cardiff University, Cardiff, UK; 2 Institute for Molecular Bioscience, University of Queensland, Brisbane, Australia; 3 Centre Cantonal Autisme, Centre Hospitalier Universitaire Vaudois, University of Lausanne, Lausanne, Switzerland; 4 Service de Génétique Médicale, Centre Hospitalier Universitaire Vaudois, Lausanne, Switzerland; 5 Departments of Pediatrics and Medicine, Columbia University, New York, NY USA; 6 Simons Foundation, New York, NY USA; 7 Department of Psychiatry, University of Washington, Seattle, WA USA; 8 Department of Pediatrics, Baylor College of Medicine, Houston, TX USA; 9 Neurodevelopmental Disorders Phenotyping Program, Divisions of Developmental Medicine and Genetics, Program in Genomics, Children’s Hospital Boston, Harvard Medical School, Boston, MA USA; 10 Division of Psychiatry, Children’s Hospital Boston, Boston, MA USA; 11 Cambridge Institute for Medical Research, University of Cambridge, Cambridge, UK; 12 Behavioural and Brain Sciences Unit, Institute of Child Health, University College London, London, UK

## Abstract

Deletion and duplication of 16p11.2 (BP4–BP5) have been associated with an increased risk of intellectual disability and psychiatric disorder. This is the first study to compare the frequency of a broad spectrum of psychiatric disorders in children with 16p11.2 deletion and duplication. We aimed to evaluate (1) the nature and prevalence of psychopathology associated with copy number variation (CNV) in children with 16p11.2 by comparing deletion and duplication carriers with family controls; (2) whether deletion and duplication carriers differ in frequency of psychopathology. 217 deletion carriers, 77 deletion family controls, 114 duplication carriers, and 32 duplication family controls participated in the study. Measures included standardized research diagnostic instruments. Deletion carriers had a higher frequency of any psychiatric disorder (OR = 8.9, *p* < 0.001), attention deficit hyperactivity disorder (ADHD) (OR = 4.0, *p* = 0.01), and autism spectrum disorder (ASD) (OR = 39.9, *p* = 0.01) than controls. Duplication carriers had a higher frequency of any psychiatric diagnosis (OR = 5.3, *p* = 0.01) and ADHD (OR = 7.0, *p* = 0.02) than controls. The prevalence of ASD in child carriers of deletions and duplications was similar (22% versus 26%). Comparison of the two CNV groups indicated a higher frequency of ADHD in children with the duplication than deletion (OR = 2.7, *p* = 0.04) as well as a higher frequency of overall psychiatric disorders (OR = 2.8, *p* = 0.02) and psychotic symptoms (OR = 4.7, *p* = 0.02). However, no differences between deletion and duplications carriers in the prevalence of ASD were found. Both deletion and duplication are associated with an increased risk of psychiatric disorder, supporting the importance of early recognition, diagnosis, and intervention in these groups.

## Introduction

Copy number variations (CNVs) in 16p11.2 (deletion and duplication) between break points 4 and 5 (BP4–BP5) (600 kb, chr16; 29.6–30.2 mb-HG19) occur at a frequency of ~3 in 10,000^1^. Around 71% of the 16p11.2 deletions occur de novo while the majority of the 16p11.2 duplications (70%) are familial^2^. This is consistent with the notion that 16p11.2 deletions have a greater impact on functioning, resulting in reduced fecundity^3^. Both 16p11.2 deletion and duplication have been associated with the risk for autism spectrum disorder (ASD). Evidence comes from studies of individuals with ASD^4–11^ with a meta-analysis of 3613 cases reporting a prevalence of 0.50% (95%CI: 0.31–0.82%) for the 16p11.2 deletion and of 0.25% (96%CI: 0.14–0.56%) for the duplication^12^. The 16p11.2 duplication, but not deletion, has been linked to risk of schizophrenia in a meta-analysis of 16,772 cases reporting a prevalence of 0.35% (95%CI: 0.27–0.45%) in cases compared to 0.03% in controls (95%CI: 0.02–0.05%)^13^. This has been supported by comparisons of schizophrenia patients and controls^13^ and parent–proband trios^14^. The effect of these CNVs on mean IQ is a decrease of approximately two standard deviations for the deletion and one standard deviation for the duplication^2,15,16^. Apart from ASD, studies of 16p11.2 deletion carriers indicate high frequencies of attention deficit hyperactivity disorder (ADHD) (19%^15^ to 38%^17^), anxiety disorders (6%^15^ to 25%^18^), mood disorders (15%^18^), oppositional defiant disorder (ODD), and other disruptive disorders (13%^15^ to 39%^3^). For carriers with the duplication, high rates of ADHD (30%^3^) have been reported.

Interestingly, these chromosomal rearrangements have been associated with mirrored physical phenotypic effects. The 16p11.2 deletion has been associated with the risk of morbid obesity and large head circumference, while the 16p11.2 duplication has been associated with low body mass index (BMI) and small head circumference^2,18,19^. Taking into account evidence suggesting links between small head circumference and schizophrenia^20,21^ and large head circumference with autism^22,23^, it has been suggested that these mirrored phenotypic effects (overgrowth versus undergrowth phenotype) extend to include psychopathology^24^, although this has not yet been formally evaluated. More specifically, it has been suggested that differences in gene dosage associated with a single set of genes within 16p11.2 (deletion) versus three copies (duplication) may mediate risk of ASD versus schizophrenia^25,26^. The 16p11.2 CNV has been presented as an example of this theory, such that deletion carriers may be at increased risk of ASD in particular, whilst duplication carriers are at risk of ASD and schizophrenia^2,18,26^.

Despite strong evidence that 16p11.2 deletions and duplications are associated with an increased risk of psychiatric conditions, the majority of studies are based on limited and non-systematic assessments (e.g., medical records). Although informative, such records are less suited to systematic evaluation of large samples, because of differences between medical centers and clinicians in diagnostic methods used and clinical documentation. With the exception of schizophrenia and ASD, previous studies have been based on relatively small samples, without the availability of control samples to allow comparisons.

Here, we report the largest study to date using detailed systematically performed psychiatric assessments to determine the nature and prevalence of psychopathology in children with deletion or duplication of 16p11.2. We hypothesized that children with 16p11.2 deletion and duplication have a higher frequency of psychiatric disorder than family controls. We also hypothesized that deletion carriers would be more severely affected than duplication carriers, based on findings that the deletion is more likely to arise de novo, whereas the duplication is more frequently inherited. Finally, given the evidence that the 16p11.2 CNV is associated with a mirror phenotype of head and body size, we hypothesized that the children with 16p11.2 deletion have a higher frequency of ASD compared to those with 16p11.2 duplication while children with 16p11.2 duplication have a higher frequency of psychotic symptoms than those with 16p11.2 deletion.

## Materials/subjects and methods

### Sample

This study combined child samples (age <18 years old) recruited through Medical Genetics clinics and collected in Europe (EU) and the US (Table 1). The samples were provided from the ECHO study, the 16p11.2 European Consortium, the IMAGINE-ID study, and the Simons VIP Consortium (Table 2). The sample characteristics of the ECHO study, 16p11.2 European Consortium, and the Simons VIP Consortium have been described elsewhere^2,15^. The study methodology and sample assessment of the UK ECHO study and IMAGINE-ID project are identical. A subset of these families (i.e., from the Simon’s VIP cohort) has been described in prior studies in terms of their IQ, ASD, and schizophrenia diagnoses^2,27^.

**Table 1:** Description of 16p11.2 cohort, carrier groups, and controls

Cohort by carrier group	Sample size	Male (%)	Age, mean (SD)	De novo (%)	Inherited
**16p11.2 deletion**					
**European**					
Carriers	101	65 (64%)	9.5 (3.1)	45 (56%)	36 (44%)
Controls	22	16 (73%)	9.8 (3.1)		
**US**					
Carriers	116	61 (53%)	8.5 (3.3)	67 (74%)	23 (26%)
Controls	55	20 (36%)	9.8 (4.0)		
**16p11.2 duplication**					
**European**					
Carriers	46	30 (65%)	9.0 (3.3)	9 (24%)	29 (76%)
Controls	7	5 (71%)	10.6 (5.7)		
**US**					
Carriers	68	35 (52%)	8.1 (3.7)	9 (16%)	46 (84%)
Controls	25	13 (52%)	8.1 (3.9)		

Counts of de novo and inherited do not add up to 100% because a number of carriers were of unknown status. Percentages are estimated not taking into account individuals with unknown status

**Table 2:** Measures used in the European and United States cohorts

Cohort	Studies included	Measures
European (EU)	ECHO	CAPA & ADI-R
	IMAGINE-ID	CAPA & ADI-R
	16p11.2 European Consortium	Clinical interviews & the Conners CBRS for children, ADI-R, and ADOS
United States (US)	Simons VIP Consortium	DISC and clinical observations for all subjects in combinations with IQ testing and supporting measures (SCL-90, BAPQ, SRS), ADI-R, and ADOS

*ECHO* the Cardiff University ExperienCes of people witH cOpy number variants (ECHO) study (see http://medicine.cf.ac.uk/psychological-medicine-neuroscience/areas-research/copy-number-variant-research/research-projects/), *IMAGINE-ID* intellectual disability and mental health: assessing genomic impact on neurodevelopment (see http://www.imagine-id.org/), *Simons VIP Consortium* the Simons Variation in Individuals Project (VIP) Consortium (see https://www.simonsvipconnect.org/), *CAPA* Child and Adolescent Psychiatric Assessment^38^, *Conners CBRS* Conners Comprehensive Behavior Rating Scales^39^, *DISC* Diagnostic Interview Schedule for Children^40^, *ADI-R* the Autism Diagnostic Interview—Revised^41^, *ADOS* the Autism Diagnostic Observation Schedule^41^, *SCL-90* Symptom Checklist—90^42^, *BAPQ* Broad Autism Phenotype Questionnaire^43^, *SRS* Social Responsiveness Scale^44^

Carrier status for the 16p11.2 deletion or duplication was confirmed for all individuals through clinical chromosome microarrays, medical records and/or confirmation in a research laboratory. All participants had a CNV in 16p11.2 BP4–BP5 region, excluding those in the adjacent region. Family members (siblings) who did not carry the CNV participated as family controls (controls) (Table 1). To assess potential recruitment bias in children, as a supplementary analysis, we further divided the carriers into (a) probands (individuals who first came to the attention of medical services) and (b) relative carriers (i.e., their siblings who were identified and diagnosed following CNV diagnosis of the proband) and compared the frequencies of psychiatric diagnoses between these two groups. Current analyses included individuals ≥3 years old in order to standardize diagnoses, especially for ASD, in very young children^15^.

The study was approved by the appropriate local ethics committees and institutional review boards. Each participant and his or her caregiver, when appropriate, provided informed written consent/assent to participate prior to recruitment.

### Psychiatric assessments

Assessments were performed using research diagnostic instruments as previously reported^2^ under the supervision of experienced and licensed clinicians who gave the best estimate clinical and research DSM-IV-TR (Table 2). Only diagnoses that were available for all participating sites were examined. Only 2% of children with deletion and duplication were diagnosed with any syndromal psychotic disorder versus 0% of controls. Therefore, psychotic symptoms were examined instead.

IQ scores have already been reported for this sample^2^. We present the full-scale IQ scores and comparisons of psychopathology for subgroups of carriers with and without intellectual disability (ID, as defined by IQ ≤ 70).

### Statistical analysis

Data analysis was conducted using Stata (version 13)^28^. Descriptive statistics were calculated to assess the frequency of psychiatric disorder. Random effects logit models were performed to determine whether group status (e.g., probands versus controls) (independent variable) was associated with an increased risk for psychiatric disorder (dependent variable: 0 = no diagnosis, 1 = diagnosis present) while accounting for familial clustering (i.e., the family that each individual belongs to). Odds ratios (ORs) and 95% confidence intervals (95%CIs) were derived and adjusted for age, sex, and cohort status (i.e., EU versus US)^2^. In cases where the maximum likelihood estimates tended to infinity (i.e., rare outcomes/zero values), Firth’s method^29^ was used^30^. To assess recruitment bias, we repeated these analyses comparing probands versus relative carriers. In order to reduce the number of statistical comparisons, anxiety disorders were included in the analyses as a summary variable (i.e., any anxiety disorder). We tested the associations between psychopathology and gender, inheritance status and ID using the phi coefficient.

## Results

Table 1 shows the descriptive statistics of the sample by cohort status (i.e., EU and US).

### Deletion carriers versus family controls

**Fig. 1:**
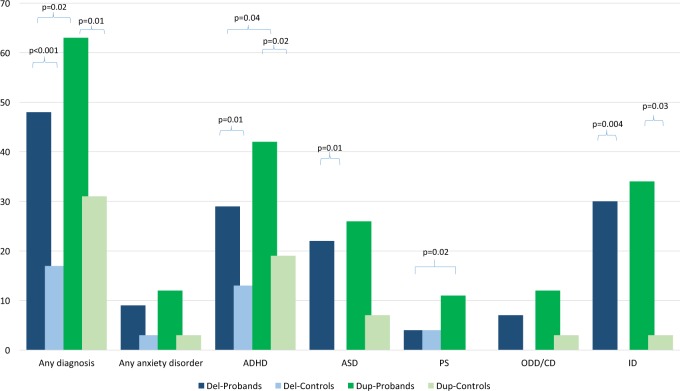
Frequency of psychiatric diagnoses, psychotic symptoms, and intellectual disability in children with 16p11.2 deletion and duplication. ADHD attention deficit hyperactivity disorder, ASD autism spectrum disorder, ID intellectual disability, ODD/CD oppositional defiant disorder/conduct disorder, PS psychotic symptoms

217 carriers and 77 controls were included in the analyses. 48% of the carriers met criteria for at least one psychiatric disorder (Table 3, S-Table 1, Fig. 1). This prevalence was higher than for controls (17%, OR = 8.9, *p* < 0.001). The most prevalent diagnosis in the carriers was ADHD (29%). This prevalence was higher than in controls (13%, OR = 4.0, *p* = 0.01). ASD was the second most common diagnosis in carriers (22%), which was higher than in controls (0%, OR = 39.9, *p* = 0.01). ID was also more frequent among carriers (30%) than controls (0%, OR = 58.7, *p* = 0.004). No significant differences were found in the prevalence of anxiety disorders, ODD/CD, and psychotic symptoms in deletion carriers compared to controls. Male children with deletions were more likely than females to be diagnosed with any psychiatric disorder, ASD and ID, and individuals with inherited deletion were more likely to have ID (S-Table 2).

**Table 3:** Psychiatric diagnoses, psychotic symptoms, and intellectual disability in children by 16p11.2 status

CHILDREN	Deletion			
	Carriers (*N* = 217)	Controls (*N* = 77)	Odds ratio (95%CI)	*p*
	*N* (%)	*N* (%)		
**Any diagnosis**	**105 (48)**	**13 (17)**	**8.9 (2.9–27.3)**	**<** **0.001**
**Any anxiety disorder**	20 (9)	2 (3)	3.0 (0.7–13.4)	0.16
**ADHD**	**63 (29)**	**10 (13)**	**4.0 (1.3–11.9)**	**0.01**
**ASD**	**41 (22)**	**0 (0)**	**39.9 (2.4–660.5)**	**0.01**
**Psychotic symptoms**	5 (4)	2 (4)	0.6 (0.1–2.7)	0.46
**ODD/CD**	15 (7)	0 (0)	9.3 (0.5–159.2)	0.12
**ID** ^a^	**61 (30)**	**0 (0)**	**58.7 (3.5–973.1)**	**0.004**

	Duplication			
	Carriers (*Ν* = 114)	Controls (*N* = 32)	Odds ratio (95%CI)	*p*
	*N* (%)	*N* (%)		
**Any diagnosis**	**72 (63)**	**10 (31)**	**5.3 (1.6–17.1)**	**0.01**
**Any anxiety disorder**	14 (12)	1 (3)	2.6 (0.3–23.6)	0.38
**ADHD**	**48 (42)**	**6 (19)**	**7.0 (1.4–35.9)**	**0.02**
**ASD**	26 (26)	3 (7)	4.5 (0.7–27.3)	0.10
**Psychotic symptoms**	7 (11)	0 (0)	2.2 (0.10–49.2)	0.61
**ODD/CD**	14 (12)	1 (3)	5.1 (0.6–46.0)	0.14
**ID** ^a^	**36 (34)**	**1 (3)**	**56.7 (1.5–2193.2)**	**0.03**

	Duplication versus deletion	
	Odds ratio (95%CI)	*p*
**Any diagnosis**	**2.8 (1.2–6.7)**	**0.02**
**Any anxiety disorder**	2.0 (0.9–4.5)	0.09
**ADHD**	**2.7 (1.0–7.1)**	**0.04**
**ASD**	1.4 (0.6–3.0)	0.44
**Psychotic symptoms**	**4.7 (1.3–17.8)**	**0.02**
**ODD/CD**	2.1 (1.0–4.7)	0.06
**ID** ^a^	1.8 (0.7–4.5)	0.19

Not all individuals had complete data on all diagnoses. Bold indicates *p* < 0.05. ID was not included in the overall rates of “any diagnosis”. The percentages represent the proportion of individuals with available diagnoses

*ADHD* attention deficit hyperactivity disorder, *ASD* autism spectrum disorder, *ODD/CD* oppositional defiant disorder/conduct disorder

^a^Defined by IQ ≤ 70.

### Duplication carriers versus family controls

114 carriers and 32 controls were included in the analyses. 63% of the carriers had at least one psychiatric disorder (Table 3, S-Table 1, Fig. 1). This frequency was higher than for controls (31%, OR = 5.3, *p* = 0.01, Table 3). ADHD was the most common diagnosis in the carriers (42%) and was elevated compared to controls (19%, OR = 7.0, *p* = 0.02). There were no differences in the prevalence of anxiety disorders, ASD, ODD/CD, and psychotic symptoms between carriers and controls. ID was higher in carriers (34%) than controls (3%, OR = 56.7, *p* = 0.03). Males were more likely to be diagnosed with ODD/CD, and individuals with ID to be diagnosed with ASD (S-Table 2).

### Deletion versus duplication carriers

Duplication carriers had higher frequencies of any diagnosis (OR = 2.8, *p* = 0.02), ADHD (OR = 2.7, *p* = 0.04) and psychotic symptoms (OR = 4.7, *p* = 0.02) than deletion carriers. No other differences were found (Table 3).

### Assessment of potential recruitment bias

No differences were found between probands and relative carriers with the deletion in the prevalence of psychiatric disorders (S-Table 3). However, probands with the duplication had higher frequencies of any psychiatric disorder than relative carriers (OR = 3.9, *p* = 0.01).

## Discussion

### Psychiatric disorders in children with 16p11.2 deletion and duplication

Our findings indicate that the mental health consequences of the 16p11.2 deletion and duplication are broad, similar to findings in other CNVs (e.g., 22q11.2 deletion syndrome^31,32^).

ASD frequencies were higher in deletion carriers when compared to controls. In addition, almost half of the carriers with either the deletion or duplication were diagnosed with at least one psychiatric diagnosis, a frequency much higher than for non-carriers. This is similar to previously reported frequencies in medical records-based studies of individuals with 16p11.2 deletion^33^ and duplication^3^. ADHD was the most commonly diagnosed disorder affecting between 30 and 40% of deletion and duplication carriers, similar to the frequencies previously reported^3,15,17,18^. ID was present in about a third of deletion and duplication carriers and was also much more common than amongst controls. The frequency of anxiety and ODD/CD disorders and psychotic symptoms did not differ between carriers and controls, consistent with the previous studies^15,18^.

Previous studies in population-based samples of CNV carriers have found high frequencies of cognitive impairments and psychiatric symptoms^24,34^. This indicates that these high frequencies are not attributable to recruitment bias but are also representative of unselected CNV populations. Indeed, we generally did not find evidence of ascertainment bias effects on phenotypic assessment, as the frequencies of psychiatric diagnoses did not differ between probands and relative carriers. The only exception was our finding of higher frequencies of any psychiatric diagnoses in child duplication compared to relative carriers.

Although we found evidence for differences in the prevalence of psychotic symptoms, we did not find evidence for differences in the prevalence of ASD between 16p11.2 deletion and duplication carriers, for which we had more power. Thus, our findings do not provide support of a mirror phenotype for a psychiatric disorder. More generally, both deletion and duplication showed significant and overlapping psychopathology and any differences were of degree rather than suggesting contrasting mirror effects at the level of psychopathology.

Interestingly, we found associations between sex and any psychiatric disorder, ASD and ID in children with 16p11.2 deletion and ODD/CD in children with 16p11.2 duplication, indicating an increased risk for males. No other associations were found with ADHD or psychotic symptoms which agrees with a previous study that examined questionnaire responses^18^ as well as with findings related to another CNV on chromosome 22, resulting in 22q11.2 deletion syndrome^32^. These findings point to the possible negation of sex-related burden to some psychiatric disorders by these CNVs^35^. Apart from an increased risk of ASD in children with 16p11.2 duplication, psychiatric disorders did not occur more frequently in participants with ID in children with 16p11.2 deletion or duplication. Although this could reflect insufficient power, it could also indicate that these CNVs have pleiotropic effects on IQ and psychopathology^36^. Finally, there was an association between inherited status and higher risk of ID indicating that potential parental genetic and environmental effects might be negatively implicated on child intelligence. There were no other associations with inheritance status and psychiatric diagnoses, similar to a previous study^15^.

### Strengths and limitations

This is the largest study to date to perform detailed phenotypic assessments on children with 16p11.2 deletion and duplication and compare the frequency of psychopathology with familial controls as well as between deletion and duplication carriers.

Our findings may be influenced by ascertainment bias. First, although we did not recruit individuals from psychiatric services, ascertainment was based on a phenotype significant enough to trigger clinical genetic testing (e.g., developmental delay). Second, although for many phenotypes we did not find evidence for recruitment bias in our analyses, we did find higher frequencies of any psychiatric diagnoses in child duplication probands compared to child relative carriers. We have previously explained our ascertainment strategies and limitations^15,32^. It is noteworthy that there were relatively high frequencies of psychopathology in familial controls. 17% and 31% of non-carrier child relatives of individuals with deletion and duplication, respectively, met criteria for at least one psychiatric diagnosis. The frequencies of ADHD (13% for deletion and 19% for duplication) and ASD (7% for duplication) were higher than we would expect from a population-based sample (5–7% and 1%, respectively). Potential explanations could be increased background environmental risk (e.g., the effect of family psychopathology), or perhaps the presence of polygenic or other additional genetic background effects that could also be a result of assortative mating. Irrespectively, our findings indicate that carriers of deletion or duplication of CNV at 16p11.2 who come to the attention of Medical Genetics services are at high risk of psychiatric disorder, and this is of importance for treating clinicians as well as the families themselves. If anything, our estimates represent an underestimate, given that the risks are elevated in our comparison sample of non-carrying familial controls.

Finally, the assessments at all sites were made by experienced clinicians and highly trained and clinically supervised psychologists. However, there were differences in the frequencies of psychiatric diagnoses and ID and although we adjusted for cohort status, we cannot exclude that potentially different diagnostic practices between the sites and countries in this study might have played a role in our findings. Further research is needed to examine psychopathology associated with these CNVs in population-based samples as well as to understand their longitudinal course.

### Clinical implications and future directions

This study clearly indicates that the phenotypic effects of the 16p11.2 deletion and duplication extend to include non-ASD psychopathology. The high frequency of psychiatric disorders, especially ADHD, in childhood indicates the need for recognition, diagnosis, and treatment early in development. Longitudinal studies are needed to examine the natural history of these disorders and whether specific types of treatment (e.g., stimulant medication for ADHD) are beneficial^37^. Not all children met criteria for a psychiatric disorder, supporting previous literature indicating that there is a spectrum of manifestations of the 16p11.2 deletions and duplications^7^, similar to other CNVs (e.g., 22q11.2DS^32^).

Future studies could examine the extent to which assortative mating plays a role in psychiatric risk in children from families where a CNV is inherited compared to where it occurs de novo. Further exploration of the extent to which background genetic as well as environmental risk factors contribute to the risk of psychiatric disorder in carriers with a de novo versus an inherited CNV is also important.

## Conclusions

We examined the frequency of psychopathology in children with 16p11.2 deletion and duplication. Our findings indicate a high frequency of psychopathology compared to familial controls and suggest the importance of recognition, diagnosis, and intervention in early development. The frequency of psychotic symptoms and ASD is similar in deletion and duplication carriers, indicating that mirrored physical effects of deletion and duplication do not extend to psychopathology.

## Supplementary information

Supplementary material
