# Correction: Psychiatric disorders in children with 16p11.2 deletion and duplication

**DOI:** 10.1038/s41398-019-0441-6

**Published:** 2019-03-05

**Authors:** Maria Niarchou, Samuel J. R. A. Chawner, Joanne L. Doherty, Anne M. Maillard, Sébastien Jacquemont, Wendy K. Chung, LeeAnne Green-Snyder, Raphael A. Bernier, Robin P. Goin-Kochel, Ellen Hanson, David E. J. Linden, Stefanie C. Linden, F. Lucy Raymond, David Skuse, Jeremy Hall, Michael J. Owen, Marianne B. M. van den Bree

**Affiliations:** 1 Division of Psychological Medicine and Clinical Neurosciences, Medical Research Council Centre for Neuropsychiatric Genetics and Genomics, Cardiff University, Cardiff, UK; 2 Institute for Molecular Bioscience, University of Queensland, Brisbane, Australia; 3 Centre Cantonal Autisme, Centre Hospitalier Universitaire Vaudois, University of Lausanne, Lausanne, Switzerland; 4 Service de Génétique Médicale, Centre Hospitalier Universitaire Vaudois, Lausanne, Switzerland; 5 Departments of Pediatrics and Medicine, Columbia University, New York, NY USA; 6 Simons Foundation, New York, NY USA; 7 Department of Psychiatry, University of Washington, Seattle, WA USA; 8 Department of Pediatrics, Baylor College of Medicine, Houston, TX USA; 9 Neurodevelopmental Disorders Phenotyping Program, Divisions of Developmental Medicine and Genetics, Program in Genomics, Children’s Hospital Boston, Harvard Medical School, Boston, MA USA; 10 Division of Psychiatry, Children’s Hospital Boston, Boston, MA USA; 11 Cambridge Institute for Medical Research, University of Cambridge, Cambridge, UK; 12 Behavioural and Brain Sciences Unit, Institute of Child Health, University College London, London, UK

**Correction to**: **Translational Psychiatry**

10.1038/s41398-018-0339-8 Published online 16 January 2019

One of the co-authors, Marianne B.M. van den Bree has had her name incorrectly abbreviated by citation manager. It was stated as “Bree MBMVD14”, but has been updated to “van den Bree, M.B.M.” in the HTML, PDF, and XML versions of this article.

