# Supplementary material for: Psychiatric disorders in children with 16p11.2 deletion and duplication
Source: Transl Psychiatry. 2019 Jan 16;9:8. doi: 10.1038/s41398-018-0339-8 (PMC6341088; doi:10.1038/s41398-018-0339-8)
Supplement: Supplementary file 1 — Supplementary material [file 41398_2018_339_MOESM1_ESM.docx]

| **S-Table 1.** Psychiatric diagnoses, psychotic symptoms and intellectual disability in children by 16p11.2 status, cohort and carrier group | | | | | | | | | | | | |
| --- | --- | --- | --- | --- | --- | --- | --- | --- | --- | --- | --- | --- |
| CHILDREN | 16p11.2 deletion | | | | | | 16p11.2 duplication | | | | | |
|  | Europe | | | United States | | | Europe | | | United States | | |
|  | Carriers | Carrier relatives | Controls | Carriers | Carrier relatives | Controls | Carriers | Carrier relatives | Controls | Carriers | Carrier relatives | Controls |
| N | 87 | 14 | 22 | 106 | 10 | 55 | 39 | 7 | 7 | 50 | 18 | 25 |
|  | Mean(SD) | Mean(SD) | Mean(SD) | Mean(SD) | Mean(SD) | Mean(SD) | Mean(SD) | Mean(SD) | Mean(SD) | Mean(SD) | Mean(SD) | Mean(SD) |
| IQ | 73.8(14.1) | 69.6(14.1) | 99.2(12.6) | 84.6(15.8) | 81.3(16.4) | 106.8(9.8) | 67.5(21.9) | 79(20.6) | 103(18.2) | 77.2(21.4) | 85.9(17.8) | 100.7(19.7) |
|  | N(%) | N(%) | N(%) | N(%) | N(%) | N(%) | N(%) | N(%) | N(%) | N(%) | %(N) | N(%) |
| Intellectual disability* | 31(41) | 8(57) | 0(0) | 20(19) | 2(22) | 0(0) | 15(44) | 2(33) | 0(0) | 16(33) | 3(17) | 1(4) |
| Any diagnosis | 41(47) | 4(29) | 3(15) | 57(54) | 3(30) | 10(18) | 29(74) | 4(57) | 2(29) | 33(66) | 6(33) | 8(32) |
| Any anxiety disorder | 13(15) | 2(14) | 0(0) | 5(5) | 0(0) | 2(4) | 7(18) | 3(43) | 0(0) | 3(6) | 1(6) | 1(4) |
| Specific phobia | 3(3) | 0(0) | 0(0) | 0(0) | 0(0) | 0(0) | 4(10) | 1(14) | 0(0) | 1(2) | 0(0) | 0(0) |
| Social Phobia | 9(10) | 0(0) | 0(0) | 1(1) | 0(0) | 0(0) | 1(3) | 2(29) | 0(0) | 0(0) | 0(0) | 1(4) |
| SAD | 4(5) | 0(0) | 0(0) | 0(0) | 0(0) | 0(0) | 0(0) | 1(14) | 0(0) | 0(0) | 0(0) | 0(0) |
| Agoraphobia | 4(5) | 1(7) | 0(0) | 0(0) | 0(0) | 0(0) | 2(5) | 1(14) | 0(0) | 0(0) | 0(0) | 0(0) |
| OCD | 2(2) | 1(7) | 0(0) | 0(0) | 0(0) | 0(0) | 0(0) | 1(14) | 0(0) | 2(4) | 0(0) | 0(0) |
| GAD | 2(2) | 1(7) | 0(0) | 4(4) | 0(0) | 2(4) | 6(15) | 2(29) | 0(0) | 0(0) | 1(6) | 0(0) |
| MDD | 0(0) | 0(0) | 0(0) | 0(0) | 0(0) | 0(0) | 0(0) | 0(0) | 0(0) | 0(0) | 0(0) | 0(0) |
| Dysthymic disorder | 1(1) | 0(0) | 0(0) | 0(0) | 0(0) | 1(2) | 1(3) | 0(0) | 0(0) | 1(2) | 0(0) | 0(0) |
| ADHD | 27(31) | 3(21) | 3(15) | 32(30) | 1(10) | 7(13) | 15(38) | 4(57) | 2(29) | 24(48) | 5(28) | 4(16) |
| ASD | 12(19) | 0(0) | 0(0) | 27(25) | 2(20) | 0(0) | 12(43) | 0(0) | 0(0) | 12(24) | 2(11) | 3(12) |
| ODD/CD | 7(8) | 2(14) | 0(0) | 6(6) | 0(0) | 0(0) | 4(10) | 3(43) | 0(0) | 7(14) | 0(0) | 1(4) |
| Psychotic symptoms | 5(10) | 0(0) | 2(13) | 0(0) | 0(0) | 0(0) | 6(40) | 0(0) | 0(0) | 1(3) | 0(0) | 0(0) |
| Any psychosis | 0(0) | 0(0) | 0(0) | 4(4) | 0(0) | 0(0) | 1(3) | 0(0) | 0(0) | 0(0) | 1(6) | 0(0) |
| Schizophrenia | 0(0) | 0(0) | 0(0) | 0(0) | 0(0) | 0(0) | 0(0) | 0(0) | 0(0) | 0(0) | 1(6) | 0(0) |
| Substance/alcohol abuse | 0(0) | 0(0) | 0(0) | 0(0) | 0(0) | 0(0) | 1(3) | 0(0) | 0(0) | 0(0) | 0(0) | 0(0) |
| Abbreviations: SAD=Separation Anxiety Disorder; OCD=Obsessive Compulsive Disorder; GAD=Generalized Anxiety Disorder; MDD=Major Depressive Disorder; ADHD=Attention Deficit Hyperactivity Disorder; ASD=Autism Spectrum Disorder; ODD/CD=Oppositional Defiant Disorder/Conduct Disorder. * defined by IQ<=70 ** no ASD diagnosis available | | | | | | | | | | | | |

**S-Table 2.** Relationship between sex, inheritance and intellectual disability with psychopathology in children with 16p11.2 deletion and duplication

| CHILDREN |  |  |  |
| --- | --- | --- | --- |
| 16p11.2 deletion |  |  |  |
| Diagnosis | Males/Females | Inheritance  (de novo/inherited) | Intellectual disability*  (No/Yes) |
| Intellectual disability | **-0.15 (p=0.03)** | **0.19 (p=0.01)** |  |
| Any diagnosis | **-0.13(p=0.05)** | -0.13 | 0.08 |
| Any anxiety disorder | -0.05 | 0.04 | 0.07 |
| ADHD | -0.05 | -0.09 | 0.02 |
| ODD/CD | -0.05 | -0.11 | 0.12 |
| Psychotic symptoms | -0.09 | 0.08 | -0.02 |
| ASD | **-0.16(p=0.03)** | -0.16 | 0.05 |
|  |  |  |  |
| 16p11.2 duplication |  |  |  |
| Diagnosis | Males/Females | Inheritance  (inherited/de novo) | Intellectual disability* |
| Intellectual disability | 0.05 | -0.04 |  |
| Any diagnosis | -0.15 | -0.18 | 0.10 |
| Any anxiety disorder | -0.00 | 0.07 | -0.20(p=0.03) |
| ADHD | -0.06 | -0.10 | -0.18 |
| ODD/CD | **-0.22(p=0.02)** | 0.19 | -0.13 |
| Psychotic symptoms | 0.18 | 0.10 | -0.04 |
| ASD | -0.10 | -0.11 | **0.45(p<0.001)** |
| In bold significant associations with p<0.05. Negative associations signify that males/inherited/no intellectual disability are at greater risk.  Abbreviations: ADHD=Attention Deficit Hyperactivity Disorder, ODD/CD=Oppositional Defiant Disorder/Conduct Disorder, ASD=Autism Spectrum Disorder, MDD=Major Depressive Disorder  * defined by IQ<=70 | | | |

**S-Table 3.** Assessing recruitment bias.

| CHILDREN | Any diagnoses | | Any anxiety | | ADHD | | ODD/CD | | Psychotic symptoms | | ASD | |
| --- | --- | --- | --- | --- | --- | --- | --- | --- | --- | --- | --- | --- |
| Comparisons | OR(95%CI) | p | OR(95%CI) | p | OR(95%CI) | p | OR(95%CI) | p | OR(95%CI) | p | OR(95%CI) | p |
| **Deletion** |  |  |  |  |  |  |  |  |  |  |  |  |
| Probands vs*. relative carriers* | 2.7(0.7-10.9) | 0.17 | 1.1(0.2-5.7) | 0.87 | 2.9(0.4-20.3) | 0.29 | 0.8(0.2-3.7) | 0.76 | 1.1(0.0-25.6) | 0.97 | 2.7(0.4-16.2) | 0.37 |
| **Duplication** |  |  |  |  |  |  |  |  |  |  |  |  |
| Probands vs*. relative carriers* | **3.9(1.3-11.5)** | **0.01** | 0.4(0.1-1.8) | 0.21 | 1.5(0.4-5.2) | 0.50 | 0.7(0.2-3.2) | 0.66 | 7.9(0.3-196.1) | 0.21 | 4.0(0.8-18.7) | 0.08 |
| ORs adjusted for cohort, age and sex; Reference group is in italics; ASD=Autism Spectrum Disorder, ADHD=Attention Deficit Hyperactivity Disorder, ODD/CD=Oppositional Defiant Disorder/Conduct Disorder, ASD=Autism Spectrum Disorder | | | | | | | | | | | | |
